# Uncoupling Aluminum Toxicity From Aluminum Signals in the STOP1 Pathway

**DOI:** 10.3389/fpls.2022.785791

**Published:** 2022-05-03

**Authors:** Léa Le Poder, Caroline Mercier, Laureline Février, Nathalie Duong, Pascale David, Sylvain Pluchon, Laurent Nussaume, Thierry Desnos

**Affiliations:** ^1^Aix Marseille Université, CEA, CNRS, BIAM, UMR 7265, SAVE, Saint Paul-lez-Durance, France; ^2^Laboratoire de Nutrition Végétale, Agroinnovation International – TIMAC AGRO, Saint-Malo, France; ^3^IRSN/PSE-ENV/SRTE/LR2T, Saint Paul-lez-Durance, France

**Keywords:** aluminum, iron, phosphate, pH, STOP1, *ALMT1*, root, Arabidopsis

## Abstract

Aluminum (Al) is a major limiting factor for crop production on acidic soils, inhibiting root growth and plant development. At acidic pH (pH < 5.5), Al^3+^ ions are the main form of Al present in the media. Al^3+^ ions have an increased solubility at pH < 5.5 and result in plant toxicity. At higher pH, the free Al^3+^ fraction decreases in the media, but whether plants can detect Al at these pHs remain unknown. To cope with Al stress, the SENSITIVE TO PROTON RHIZOTOXICITY1 (STOP1) transcription factor induces *AL-ACTIVATED MALATE TRANSPORTER1* (*ALMT1*), a malate-exuding transporter as a strategy to chelate the toxic ions in the rhizosphere. Here, we uncoupled the Al signalling pathway that controls STOP1 from Al toxicity using wild type (WT) and two *stop1* mutants carrying the *pALMT1:GUS* construct with an agar powder naturally containing low amounts of phosphate, iron (Fe), and Al. We combined gene expression [real-time PCR (RT-PCR) and the *pALMT1:GUS* reporter], confocal microscopy (*pSTOP1:GFP-STOP1* reporter), and root growth measurement to assess the effects of Al and Fe on the STOP1-ALMT1 pathway in roots. Our results show that Al triggers STOP1 signaling at a concentration as little as 2 μM and can be detected at a pH above 6.0. We observed that at pH 5.7, 20 μM AlCl_3_ induces *ALMT1* in WT but does not inhibit root growth in *stop1* Al-hypersensitive mutants. Increasing AlCl_3_ concentration (>50 μM) at pH 5.7 results in the inhibition of the *stop1* mutants primary root. Using the green fluorescent protein (GFP)-STOP1 and *ALMT1* reporters, we show that the Al signal pathway can be uncoupled from the Al toxicity on the root. Furthermore, we observe that Al strengthens the Fe-mediated inhibition of primary root growth in WT, suggesting an interaction between Fe and Al on the STOP1-ALMT1 pathway.

## Introduction

Aluminum (Al) is toxic to plants by reducing root growth and decreasing plant development. The Al-induced toxicity problem in soils only occurs at the most acidic pHs (<5) (Kochian et al., 2004, 2015). Because the speciation and solubility of Al species in water solution widely depends on the pH, and that free Al^3+^ ion is predominant under acidic conditions but absent at pH > 5.0 (Martin, 1991), Al^3+^ is often assumed to be the main toxic species of Al below pH 5. The fraction of Al^3+^ decreases with increasing pH where Al precipitates from the main Al species. Thus, many studies focusing on the effects of Al in plants are performed at acidic pH < 5, and often at pH 4.2 compatible with Al^3+^ solubility for *Arabidopsis* seedlings growing *in vitro*. It is therefore questionable whether, at a pH closer to neutrality, i.e., between > 5.5 and 6.5, plants detect Al. As Al toxicity is potentially exerted on several targets (Kochian et al., 2015), it is possible that there are distinct molecular mechanisms of Al-detection and signaling. This multiplicity of targets is a hindering factor in the search for Al’s sensing mechanisms. In *Arabidopsis*, the STOP1 transcription factor is required for the tolerance of several abiotic stresses, including both acidic pH and aluminum (Daspute et al., 2017; Koyama et al., 2021; Sadhukhan et al., 2021). The SENSITIVE TO PROTON RHIZOTOXICITY1 (STOP1) regulates the expression of several genes including *AL-ACTIVATED MALATE TRANSPORTER1* (*ALMT1)* that encodes an anion channel that exudes malate in to the rhizosphere (Iuchi et al., 2007; Sawaki et al., 2009; Tokizawa et al., 2021). Small organic acids such as malate and citrate, for example, chelate Al^3+^ ions and are key factors involved in Al tolerance mechanisms (Kochian et al., 2015). The roles of STOP1 have been independently identified by several forward genetic approaches. Originally, *stop1* mutants were isolated on the basis of their high root sensitivity to acidic pH (Iuchi et al., 2007). Subsequently, it was shown that these mutants are also highly sensitive to Al, as for the *almt1* mutants (Hoekenga et al., 2006; Kobayashi et al., 2007; Sawaki et al., 2009). But as Al sensitivity studies are always performed at acidic pH, compatible with Al^3+^ solubility, it is not always clear in these studies whether Al signaling is independent of acidic pH. Nevertheless, protons and Al exert distinct response mechanisms in plants (Shavrukov and Hirai, 2016) and studies with plant natural variation showed that tolerance to Al and H^+^ could be genetically dissociated in Arabidopsis (Ikka et al., 2007; Nakano et al., 2020). In later works, *stop1* and *almt1* mutants were isolated on the basis of improved root growth on low phosphate (−Pi) medium (which inhibits WT primary root growth) from WT background (Balzergue et al., 2017; Mora-Macias et al., 2017) or as a suppressor of the *als3* mutant that is hypersensitive to −Pi condition (Wang et al., 2019). We showed that, under −Pi condition, Fe inhibits root growth in wild-type (WT) seedlings (Svistoonoff et al., 2007; Ward et al., 2008; Muller et al., 2015). The reduction in root growth depends on the malate exuded *via* ALMT1. According to current models, exuded malate interacts with Fe^2+^ in the apoplast where redox cycling of Fe mediated by the apoplastic ferroxidase LPR1 (Low Phosphate Root1), and possibly blue-light, in a Fenton-like reaction, generate reactive oxygen species (ROS) (Muller et al., 2015; Balzergue et al., 2017; Mora-Macias et al., 2017; Zheng et al., 2019). These ROS inhibit cell expansion, at least by rapidly increasing cell wall stiffness and decreasing cell division after longer periods. All these reactions are suppressed by neutral pH (Abel, 2017) (see the schematic summary in the introduction of Mercier et al., 2021).

As Al, Fe positively regulates the stability of the STOP1 protein in root cell nuclei (Godon et al., 2019; Fang et al., 2020; Tokizawa et al., 2021). Interestingly, the effects of Al and Fe on the stability of STOP1 are dependent on acidic pH (Godon et al., 2019). Thus, at pH > 5.5–5.8 Fe and Al are less effective in stabilizing STOP1. Al and Fe, therefore, act under similar conditions (Pi, pH) and have similar targets (STOP1 signaling) allowing them to act on the plant. Al and Fe positively regulate STOP1 abundance in roots by reducing its degradation by the proteasome (Godon et al., 2019). Indeed, the ubiquitin ligase RAE1 (REGULATION OF ALMT1 EXPRESSION1) and RAH1 (RAE1 homolog1), the SUMO (small ubiquitin-related modifier) E3 ligase SIZ1 (SAP and MIZ1 domain-containing ligase1), and the SUMO protease ESD4 (EARLY IN SHORT DAYS4) are regulators of STOP1 abundance (Godon et al., 2019; Zhang et al., 2019; Fang et al., 2021a,b; Xu et al., 2021), and STOP1 is SUMOylated *in planta* (Fang et al., 2020). Furthermore, hyperrecombination protein 1 (HPR1) and TEX1, two proteins of the conserved THO/TREX complex involved in transcription, messenger RNA (mRNA) processing, and nucleocytoplasmic export of transcripts also reduce STOP1 abundance (Guo et al., 2020; Zhu et al., 2021). Apart from these proteins, which are not specific to STOP1 signaling, we do not know the steps upstream of STOP1 in this pathway, in particular, the Al-sensing mechanism. Refining growth conditions modulating STOP1 activity would help identify other regulatory steps of the signaling.

In this work, we tested whether *Arabidopsis* seedlings perceive Al under *in vitro* culture conditions not conducive to its toxicity (pH > 5 and low concentration), whether acidic pH induces *ALMT1* independently from Al and whether Al can interfere with the effect of Fe on root growth. We used the *pALMT1:GUS* reporter to finely monitor the sensing of Al and Fe by seedlings (Balzergue et al., 2017; Godon et al., 2019). Our work shows that Al can be detected at pH > 5.7 and that Al signal pathway can be uncoupled from its toxicity. The use of the green fluorescent protein (GFP)-STOP1 and *pALMT1:GUS* reporters combined with a specific agar with low-Al and Fe content enabled us to distinguish between Al and H + stress-inducing conditions. Finally, we observed that Al interferes with root response to Fe.

## Materials and Methods

### Plant Material

The Arabidopsis leaky allele *stop1*^33^ and the null allele *stop1*^127^ are both in a Col^*er*105^ genetic background and come from an ethyl methanesulfonate mutagenesis screen (Balzergue et al., 2017). The *pALMT1:GUS* reporter (*GUS*, *uidA* gene encoding β-glucuronidase) (Godon et al., 2019) was introgressed by crossing in the *stop1*^33^ and *stop1*^127^ mutant backgrounds (Mercier et al., 2021). The *pSTOP1:GFP-STOP1_#B10_* construct used was described in Balzergue et al. (2017).

### Plant Growth

Plants were grown in agar in long (16 h photoperiod) or short days (8 h photoperiod), at 22°C (day)/21°C (night).

### Seedling Growth

The seeds were surface-sterilized for 2 min in a solution containing 70% ethanol and 0.05% sodium dodecyl sulfate, and washed twice with 96% ethanol.

The nutrient solution contained 0.47 mM MgSO_4_, 2.1 mM NH_4_NO_3_, 1.89 mM KNO_3_, 0.67 mM CaCl_2_, 0.5 μM KI, 0.79 mM H_3_BO_3_, 10 μM MnSO_4_, 5 μM ZnSO_4_, 1 μM Na_2_MoO_4_, 0.1 μM CuSO_4_, 0.1 μM CoCl_2_, 5 g L^–1^ sucrose. The agar (8 g.L^–1^) for plates was from Sigma-Aldrich (A7921 Lot BCBZ7284, see Mercier et al., 2021 for elemental composition). The agar media were supplemented with 10 μM and 500 μM KH_2_PO_4_ for low-phosphate and rich-phosphate conditions, respectively. The media were buffered with 3.4 mM 2-(N-morpholino) ethanesulfonic acid (MES) for pH range from 5 to 6, and with piperazine-N,N′-bis 2-ethanesulfonic acid (PIPES) 3.4 mM from pH 6 to 7. The pre-culture was performed in rich-phosphate conditions (P_500_) and 10 μM FeCl_2_ to avoid additional chlorotic stress. After transfer on exposure media, the assessment of Fe and Al effects was performed in low-phosphate condition (P_10_). Fe (FeCl_2_) and Al (AlCl_3_) were added independently to the growth media.

### Hydroponic Culture

Hydroponic culture was performed in MS/10 nutritive solution, 0.5% sucrose with 3.4 mM buffer, Homo-Pipes (pH 4.4) or MES (pH5–6.2). Plants were grown in long days (16 h photoperiod) for 3 days and transferred in a fresh medium for 12 h for stress assessment.

### Speciation of Aluminum

The speciation of Al was simulated with the JChess 2.0 software (van der Lee and De Windt, 1999), with the database released in 2004 in the framework of the Common Thermodynamic Database Project (van der Lee and Lomenech, 2004) and updated afterward (Denison and Garnier-LaPlace, 2005; Lofts et al., 2015; Vercouter et al., 2015; Février et al., 2021). In this database, thermodynamic complexation constants for Al were imported from the thermo.com database developed by the Lawrence Livermore National Laboratory. Equilibrium reactions with their associated thermodynamic constants were presented in Supplementary Table 1. They include the formation of mononuclear hydroxyl-Al and polynuclear hydroxyl-Al species, sulfate and phosphate Al-complexes, as well as solid phases such as Gibbsite, a poorly soluble Al hydroxide [Al(OH)_3_], and Alunite, a hydroxylated Al potassium sulfate mineral [KAl_3_(SO_4_)_2_(OH)_6_].

Simulations of Al speciation were performed for each experiment. The entire chemical composition of the culture medium was considered as input in the modeling, with the exception of Homo-Pipes, MES, and agar, which are supposed to be inert toward Al speciation (Parent and Campbell, 1994).

### Green Fluorescent Protein-Fluorescence

Plants were grown for 5 days on P_500_Fe_10_ without AlCl_3_ added to the medium then transferred for 4 h on the described media in low phosphate condition (P_10_) before observation through confocal microscopy. The GFP-fluorescence was performed as in Godon et al. (2019). Images were collected on a Zeiss LSM780 confocal microscope (Carl Zeiss)^1^ using an ×40 water objective. The GFP was excited with an Ar ion laser (488 nm). Emitted light was collected from 493 to 538 nm for GFP, using the MBS 488 filter. All nuclei were imaged using the same conditions of gain, offset, and resolution. The quantification of GFP fluorescence was carried out as follows: a Z-stack of six images (separated by a distance of 2.0 μm) imaged representative nuclei on the surface of the root. Images were acquired in 12 bits using Zen black software (SP2 v.11.0, 2012, Carl Zeiss), then converted to a maximum projection image. The average nuclear fluorescence intensities were quantified using Image J by applying a mask for nuclei detection. These nuclei formed regions of interest (ROI) in which the GFP mean intensity was quantified. The GFP fluorescence intensity of minimum 15 nuclei per plant was averaged. Six plants per condition were assessed. The average of each plant was used for statistical analysis using the Kruskal-Wallis test.

### Real-Time PCR

Plants were grown for 10 days before transfer for 24 h. The roots were collected to perform RT-PCR. Total RNA was extracted from whole roots using the RNeasy Plant Mini Kit (Qiagen, France) and treated with the RNase-free DNase Set (Qiagen, France) according to the manufacturer’s instructions. Reverse transcription was performed on 400 ng total RNA using the SuperScript VILO DNA Synthesis Kit (Invitrogen). Real time quantitative reverse transcription PCR (qRT–PCR) was performed on a 480 LightCycler thermocycler (Roche) using the manufacturer’s instructions with Light cycler 480 sybr green I master (Roche) and with primers listed in Supplementary Table 2. We used tubulin (AT5G62690) as a reference gene for normalization.

### GUS Histochemical Staining

The GUS staining of Arabidopsis seedlings was conducted as previously described (Balzergue et al., 2017) for different time specified in each result.

## Results

In this work, we have selected a specific agar medium characterized by a low content in P, Fe, and Al. The low amount of these elements allowed us to assess the specific effect of each element. However, to avoid severe phosphate starvation, 10 μM KH_2_PO_4_ was added to the medium.

### Aluminum Triggers *AL-ACTIVATED MALATE TRANSPORTER1* Expression at pH 6.5

**FIGURE 1:**
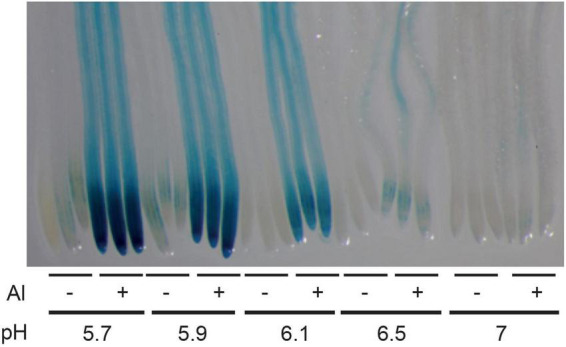
Effect of pH and aluminum (Al) on the expression of *ALMT1* in the root tip. Four day-old WT seedlings (carrying the *pALMT1:GUS* reporter) were grown on a phosphate-rich medium and transferred on the indicated P_10_Fe_0_ medium supplemented or not with 20 μM AlCl_3_ and buffered at the indicated pH. After 3 days a GUS staining was performed on roots for 1 h. Three representative root tips are shown per condition.

As described in the introduction, it is assumed that at pH > 5.5, Al has low toxic effects on plants and on the STOP1-ALMT1 pathway. In order to test whether the expression of *ALMT1* is induced by Al at pH > 5.5, 4 days-old WT seedlings carrying the *pALMT1:GUS* reporter were transferred for 3 days on growth media supplemented with 20 μM AlCl_3_ and buffered at pH from 5.7 to 7. As a negative control, some seedlings were transferred on the same media but not supplemented with AlCl_3_. As shown on Figure 1, on control conditions without AlCl_3_, no or low GUS staining is detected on the root tip. On media supplemented with AlCl_3_, the GUS staining is visible in the root tip of seedlings transferred up to a pH 6.5. No staining is observed at pH 7.0.

### Seedlings Detect Low Concentrations of AlCl_3_ at pH 5.7

**FIGURE 2:**
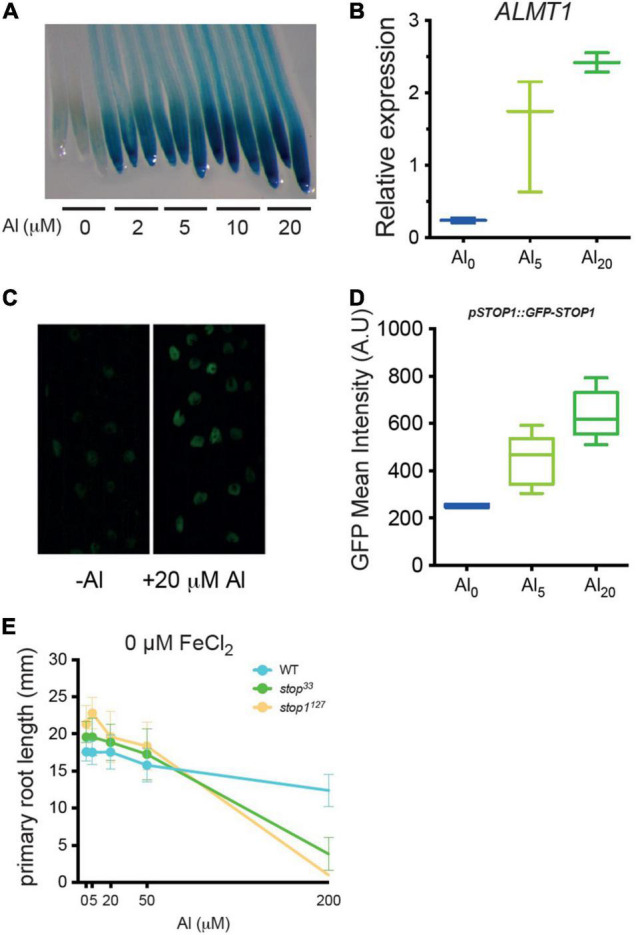
Low concentrations of AlCl_3_ at pH5.7 are sufficient to induce *ALMT1* through the accumulation of STOP1 in root nuclei. **(A)** Dose-response of AlCl_3_ on *ALMT1* expression. Four day-old WT seedlings (carrying the *pALMT1:GUS* reporter) were grown on phosphate-rich medium and transferred on the indicated P_10_Fe_0_, pH 5.7 medium supplemented with the indicated concentrations of AlCl_3_. After 3 days a GUS staining was performed on roots for 1 h. Three representative root tips are shown per condition (see Supplementary Figure 1A for a *stop1*^127^ null mutant grown in the same conditions). **(B)** Relative expression (RT-PCR) of *ALMT1* in presence of 5 and 20 μM AlCl_3_ at pH 5.7 in 10 days old roots (see section “Materials and Methods). *n* = 3 experiments, here one of the three independent experiments is shown. **(C)** Representative confocal pictures (×40) of 5 days old *pSTOP1:GFP-STOP1* seedlings were grown on P_500_Fe_10_ pH5.7 medium and transferred 4 h on the indicated medium pH5.7. **(D)** Measurement of green fluorescent protein (GFP) fluorescence (A.U.) (see section “Materials and Methods) on the indicated medium. The multiple comparisons, Kruskal-Wallis non-parametric test was performed with the application of the Dunn’s correction (*n* = 6). One of the three independent experiments is shown. **(E)** Al dose-response curve of root growth. Four day-old WT, *stop1*^33^ and *stop1*^127^ seedlings were grown on phosphate-rich medium (P_500_) and transferred on the indicated P_10_Fe_0_, pH 5.7 medium supplemented with the indicated concentrations of AlCl_3_. After 3 days the primary root lengths were measured. Mean ± SD (*n* = 13–16 seedlings).

In the literature, it is widely described that *ALMT1*-induced expression results directly from the activity of the STOP1 transcription factor. Here, we assessed the expression of *pALMT1:GUS* marker in WT seedlings grown at pH 5.7, supplemented with concentrations of AlCl_3_ ranging from 0 to 20 μM (Figure 2A). The leaky *stop1*^33^ allele displays a decreased *ALMT1* expression resulting from a partial STOP1 activity while the *stop1*^127^ null allele does not show GUS staining as a consequence of the absence of STOP1 (Balzergue et al., 2017; Mercier et al., 2021). In WT, we observed that the intensity of the staining increases with the concentration of Al. In these conditions, *ALMT1* is induced at Al concentration as low as 2 μM AlCl_3_, showing that this reporter is highly sensitive. Furthermore, this activation of *ALMT1* depends on STOP1 because in a *stop1*^127^ null mutant no GUS staining is detected independently from the Al concentration added (Supplementary Figure 1A). To check the absence of effect from the agar-solidified medium, a similar experiment was performed in a hydroponic solution. Supplementary Figure 1B shows the induction of *ALMT1* when 20 μM AlCl_3_ was added in the medium at pH 5.7. The *pALMT1:GUS* is a sensitive visual reporter, however, the *ALMT1* expression was quantified using RT-PCR in the whole root. The relative expression of *ALMT1* was measured in plants after 24 h transfer from phosphate-rich media to low phosphate media containing 0, 5, and 20 μM AlCl_3_. Figure 2B shows that *ALMT1* is induced when AlCl_3_ is added in the media. Furthermore, the rate of induction correlates with Al concentration, as with the *pALMT1:GUS* reporter.

It was previously shown that Al^3+^ increases the stability of the STOP1 protein in root nuclei (Godon et al., 2019). To determine whether the increased *ALMT1* induction is correlated with an increased STOP1 abundance, we used the *pSTOP1:GFP-STOP1* reporter. Five days old seedlings that grew in phosphate-rich medium were transferred for 4 h on the described media and then observed by confocal microscopy. Figure 2C shows that STOP1 accumulated in root nuclei 4 h after transfer of the seedlings on a medium supplemented with AlCl_3_. The fluorescence in the nuclei was quantified (Figure 2D). While no statistical differences were observed between 0 and 5 μM AlCl_3_ nor between 5 μM and 20 μM AlCl_3_, the STOP1 abundance was significantly different comparing the negative control and 20 μM AlCl_3_ (*p* < 0.01, Kruskal-Walis and Dunn’s multiple comparison test). These results show a correlation between Al-induced STOP1 accumulation in root nuclei and the *ALMT1* expression assessed by the GUS stainings (Figure 2A) and the RT-PCR (Figure 2B). Together, these experiments show that, at pH 5.7, low concentrations of AlCl_3_ are able to activate the STOP1-ALMT1 pathway in root seedlings.

The precedeing experiments show that Arabidopsis seedlings detect Al in conditions where it is not toxic. To assess which concentrations of Al are toxic in our growth conditions without iron (P_10_Fe_0_), we measured the length of the primary root (Figure 2E). The *stop1*^127^ null mutant and the *stop1*^33^ leaky mutant were included as Al-sensitive controls (Balzergue et al., 2017; Mercier et al., 2021). The dose-response curve shows that at 5, 20, and 50 μM AlCl_3_, the three lines were not different from their untreated controls. By contrast, at a higher concentration, 200 μM AlCl_3_, the root growth of *stop1*^127^ was blocked; the *stop1*^33^ was slightly less inhibited than *stop1*^127^ while the WT was about 80% the size of the control at 0 μM AlCl_3_. This experiment shows that at pH 5.7, Al is not toxic at concentrations ≤ 50 μM AlCl_3_, even in the hypersensitive *stop1* mutants. Combined with the previous results, these experiments demonstrate that a growth medium with agar, at pH 5.7 with as low as 2 μM AlCl_3_, is a condition uncoupling Al-sensing from Al-toxicity.

### Analysis of the Combined Effects of pH and Aluminum Concentration on Aluminum Speciation

In order to assess which Al species are present in the nutrient solution of our growth media, a simulation was performed with the JChess 2.0 software (see section “Materials and Methods”). To note, these simulations did not take account of the agar and the buffers. According to preliminary simulations, the culture medium was oversaturated with gibbsite and with alunite also at low pH (between 4.4 and 4.5) (not shown). However, although gibbsite is a poorly soluble mineral, its formation in the culture medium has been shown to be kinetically limited (Parent, 1991) and might be also impacted by the presence of agar. Therefore, precipitation of Al was discarded in the speciation simulations. In the first simulation with 20 μM AlCl_3_ at different pH, the predominant form of Al was Al^3+^ at low pH (4.4 < pH < 5.1). As soon as the pH increased above 5, the polynuclear specie Al_13_O_4_(OH)_24_^7+^ (referred to as Al_13_) increased up to 70% of the total Al. At pH 6.0 and above, the mononuclear hydroxy species—neutral Al(OH)_3(*aq)*_ and aluminate (Al(OH)_4_^–^) became dominant (Supplementary Figure 2A).

In the second simulation, we used different concentrations of Al at pH 5.7 (Supplementary Figure 2B). In contrast to the first simulation, the speciation of Al was affected by the concentration of Al. At low Al concentration, mononuclear Al hydroxyl species were the main Al species in the culture medium, with about 48% of neutral Al(OH)_3(*aq)*_ and 19% of Al(OH)_2_^+^. However, at a concentration above 10 μM of AlCl_3_, the polynuclear species Al_13_ prevailed over other Al species. At 40 μM AlCl_3_, Al_13_ represented more than 80% of Al species. To note, except Al_13_ that exceeds 10 μM the values of each other species remained below 5 μM independently from the AlCl_3_ concentrations assessed (up to 200 μM) (Supplementary Figure 2B inset). Thus, no species prevailed the others. According to these simulations, our medium supplemented with 20 μM AlCl_3_, at pH 5.7, should be toxic, at least for the Al-sensitive *stop1* mutants due to the extreme toxicity of polynuclear Al_13_ species (Kinraide, 1991). Since we do not observe toxic symptoms (Figure 2E), we infer that the agar might prevent toxicity of Al_13_, and possibly of other species.

### H^+^ and Aluminum Independently Induce the STOP1-*AL-ACTIVATED MALATE TRANSPORTER1* Pathway

**FIGURE 3:**
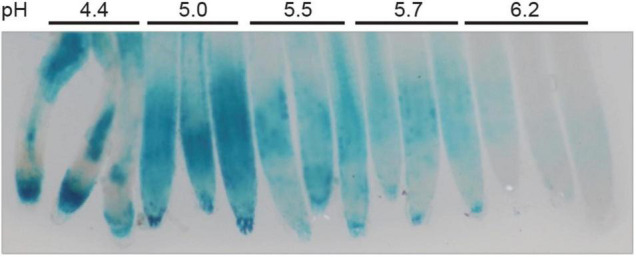
*ALMT1* is expressed under acidic pH independently from the presence of Al. Seedlings were grown in hydroponic solution at the indicated pH in phosphate-rich condition (P_500_) for 3 days. A total of 7 plants were transferred in a 30 mL fresh solution at the indicated pH for 12 h before GUS staining for 1 h.

As described in the literature, STOP1 was firstly identified as required for tolerance to acidity (Iuchi et al., 2007), then toxic Al (Sawaki et al., 2009; Kobayashi et al., 2013). However, as previously mentioned in the introduction, the speciation of Al makes it mostly studied at low pH due to its increased toxicity. As consequence, it is difficult to differentiate responses to Al from responses to low pH in these conditions. Here, our objective was to identify conditions to dissociate both mechanisms. WT plants carrying the *pALMT1:GUS* reporter were grown for 4 days on a phosphate-rich medium at the indicated pH before GUS staining (Supplementary Figure 3). This result shows that *ALMT1* expression was induced when the pH is below 5.5 in absence of Al. To note, similar results when plants were grown on a low-phosphate medium (Data not shown). Three independent experiments were performed on agar-grown plants. Although this agar was selected for its low amount of Al, the decreasing pH could increase the Al^3+^ fraction of the remaining Al in the agar, and as the *pALMT1:GUS* was shown to be highly sensitive to Al, a similar experiment was performed in hydroponics. In Figure 3, WT plants carrying the *pALMT1:GUS* reporter were grown 3 days in hydroponic medium (see section “Materials and Methods”) in phosphate-rich conditions not supplemented with Fe or Al, at the indicated pH, and then transferred in a fresh phosphate-rich medium for one more day before staining for GUS activity. The results show that, in absence of Al, *ALMT1* expression is induced at low pH. Therefore, these growth conditions uncouple the low pH-dependent *ALMT1* expression form the Al- dependent.

### Aluminum Strengthens the Iron Responses Inducing the STOP1 Pathway

**FIGURE 4:**
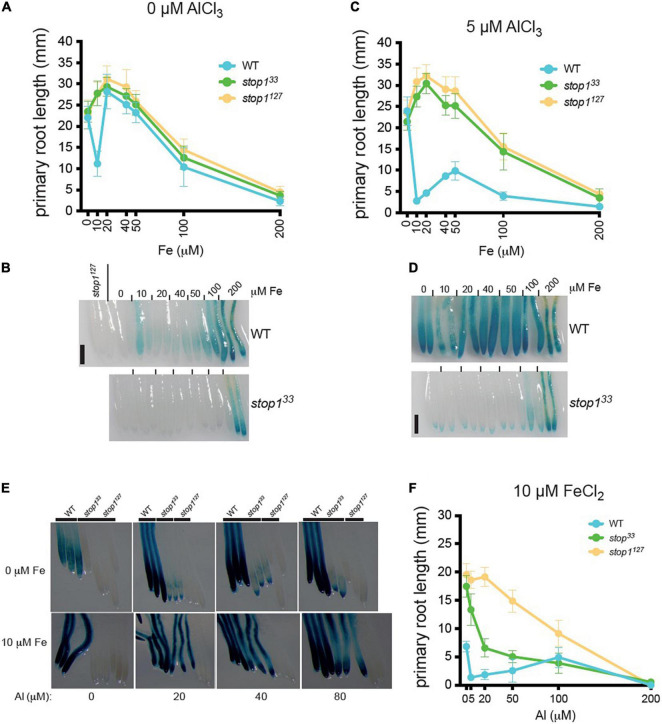
ALMT1 expression is independently activated by iron (Fe) and Al but both mechanisms’ response can interact to enhance *ALMT1* response in the root tip. **(A)** WT, *stop1*^33^ and *stop1*^127^ seedlings, carrying the *pALMT1:GUS* reporter, were grown 4 days on phosphate-rich medium (P_500_) and transferred on a P_10_ medium pH 5.7 at the indicated concentrations of FeCl_2_. After 3 days, the Δ root growth of the primary roots was measured, and **(B)** seedlings were stained for GUS activity for 1 h. As a negative control of the GUS staining, the root tip of the two *stop1*^127^ seedlings. Scales = 100 μm. Mean ± SD (*n* = 13–18 seedlings). **(C)** WT, *stop1^33^*, and *stop1*^127^ seedlings, carrying the *pALMT1:GUS* reporter, were grown 4 days on a phosphate-rich medium and transferred on a P_10_ medium with 5 μM AlCl_3_ at pH 5.7 at the indicated medium. After 3 days, the Δ root growth of the primary roots was measured, and **(D)** seedlings were stained for GUS activity for 1 h. As a negative control of the GUS staining, the root tip of the two *stop1*^127^ seedlings. Scales = 100 μm. Mean ± SD (*n* = 13–18 seedlings). **(E)** Four day-old WT, *stop1^33^*, and *stop1*^127^ seedlings (carrying the *pALMT1:GUS* reporter) were grown 4 days on a P_500_Fe_10_ medium and then transferred 3 days on low-phosphate (10 μM KH_2_PO_4_) at the indicated condition before the GUS staining for 2.5 h. Three representative root tips are shown per condition. **(F)** WT, *stop1^33^*, and *stop1*^127^ seedlings were grown 4 days on a phosphate-rich medium and transferred on P_10_Fe_10_ medium with the indicated concentrations of AlCl_3_, and the primary roots were measured. Mean ± SD (*n* = 8–14 seedlings).

We previously demonstrated that both Fe and Al trigger STOP1 signaling toward *ALMT1* expression (Godon et al., 2019) and that, under low-phosphate conditions, Fe inhibits root growth (Svistoonoff et al., 2007; Balzergue et al., 2017). Here, we tested the hypothesis that Fe and Al interfere with the expression of *ALMT1* and the root growth under low-phosphate conditions. Dose-response curve of Fe was performed on WT, *stop1*^33,^ and *stop1*^127^ seedlings (see section “Materials and Methods”). Figure 4A shows that the WT and the two mutants display similar responses, except at 10 μM FeCl_2_, where the WT is inhibited. The *stop1* mutants are insensitive to 10 μM FeCl_2_ whereas root growth of WT plants was significantly reduced. Surprisingly, the WT growth is not inhibited by 20–50 μM of FeCl_2_ and displays the same primary root length as the mutants. At concentrations above 50 μM, the root growth is inhibited similarly for the three lines. This experiment shows that the growth inhibition of the WT is restricted to a narrow range of Fe concentration (i.e., around 10 μM). Figure 4B shows the corresponding *ALMT1* expression staining to the Fe dose-response curve. In the WT we observe a slight induction of the *ALMT1* expression at 10 μM FeCl_2_. The *ALMT1* expression is increased along with the Fe concentration from 50 μM. In the *stop1^33^ leaky* mutant, the *ALMT1* expression is detected only at 200 μM Fe.

We then assessed the effect of Al on the Fe-dependent inhibition of WT root growth. The seedlings were grown 4 days on a P_500_ medium and transferred 3 days on a P_10_ medium supplemented with 0–200 μM FeCl_2_, and with 5 μM AlCl_3_ (Figure 4C). We observed that Al enhances the Fe-dependent inhibition of WT root growth under low-phosphate and at 10 μM the WT root is inhibited. Intriguingly, at intermediate concentrations of FeCl_2_ (20–100 μM) the root growth is partially restored (compared to that at 10 μM). The overall pattern of response is similar to the one observed in absence of Al (Figure 4A), but Al strengthens the inhibiting effect of the Fe on the primary root growth. At 200 μM FeCl_2_, the root growth is blocked. In these growth conditions, the expression of *ALMT1* in WT root tips is induced at each Fe concentration tested (Figure 4D). In the *stop1*^33^ mutant, *ALMT1* is slightly induced in the root cap and strongly expressed in the whole root at 200 μM Fe (Figure 4D).

This experiment shows that Al enhances both the Fe-dependent root growth inhibition and *ALMT1* expression. In addition, comparing the dose-response curves obtained with or without Al unveiled a complex effect of intermediate concentrations of Fe on root growth. Altogether the GUS staining in Figures 4B,D show that Fe and Al independently activate *ALMT1* expression in WT.

To better characterize the effect of Al on the Fe-dependent responses, we performed an Al dose-response experiment in a medium supplemented, or not, with 10 μM FeCl_2_ (Figures 4E,F). In Figure 4E (top row) in absence of Al, the WT displays a light *ALMT1* expression while no *ALMT1* induction is observed in the mutants. When 20 μM or higher concentrations of AlCl_3_ are added to the medium without supplemented Fe, *ALMT1* expression is largely induced in the WT. However, in the *stop1*^33^ leaky mutant, we observe low staining that does not increase with AlCl_3_ concentration. By contrast, when 10 μM FeCl_2_ are added in the Al-containing media, a strong *ALMT1* induction is observed in the *stop1*^33^ leaky allele (Figure 4E, bottom row). Fe and Al independently activate *ALMT1* expression in WT but not, or low *ALMT1* induction in *stop1*^33^. However, Fe and Al trigger higher *ALMT1* expression in the leaky allele. The observed staining of WT and *stop1*^33^ on Al concentration range in presence of 10μM FeCl_2_ is in correlation with the respective primary root responses (Figure 4F). The WT shows a strong primary root inhibition while the *stop1*^33^ mutant displays an intermediate root arrest compared to the null mutant stop1^127^ (Figure 4F). In these conditions, the root growth of the *ALMT1* expressing lines (WT and *stop1*^33^) is more sensitive than the *stop1*^127^ null allele. Comparison with Figure 2E indicates that WT inhibition at ≤ 50 μM Al depends on the added Fe. Of note, as in Figure 4C, a release of the WT primary root inhibition is also observed at 100 μM AlCl_3_. These results suggest that Fe and Al on *ALMT1* induction, but also root growth, are distinct signal response mechanisms as they independently induce STOP1. However, Fe and Al response mechanisms interact when both ions are combined. Based on the dose-response curve, Al strengthens the inhibitory effect of Fe on growth of primary roots. The cumulated stress up-regulate *ALMT1* in the WT but also stop1^33^ while both stresses independently applied cannot.

**FIGURE 5:**
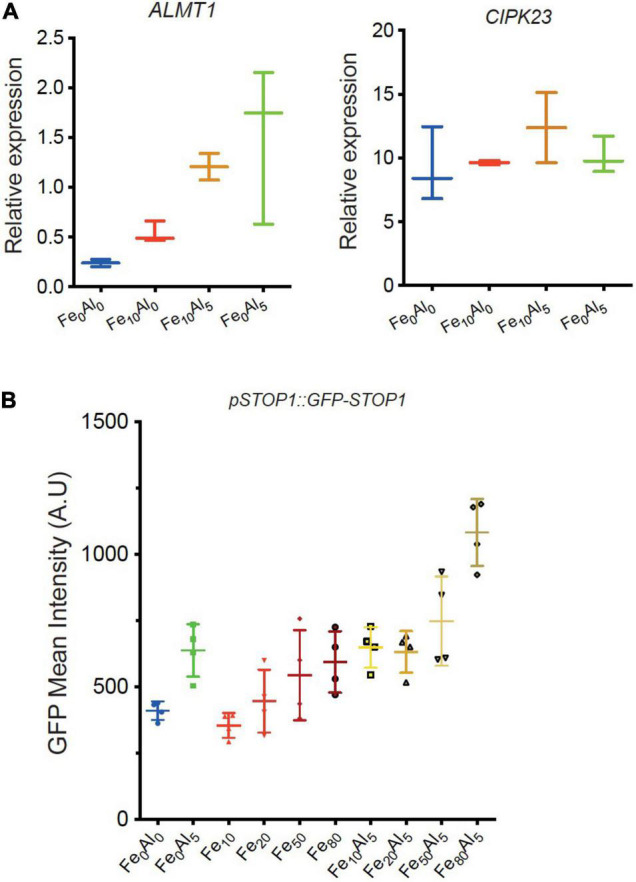
Relative expression of STOP1 downstream genes *ALMT1* and *CIPK23.* **(A)** Relative quantification (RT-PCR) of *ALMT1* and *CIPK23* STOP1 downstream genes at the indicated condition at pH 5.7 (see section “Material and Methods”), *n* = 3 experiments, one of the three independent experiments is shown. **(B)** Measurement of GFP fluorescence (A.U.) (see section “Materials and Methods”) of 5 days old *pSTOP1:GFP-STOP1* seedlings were grown on P_500_Fe_10_ pH 5.7 medium and transferred 4 h on the indicated medium pH 5.7 (*n* = 4). The multiple comparisons, Kruskal-Wallis non-parametric test was performed with the application of the Dunn’s correction. One of the three independent experiments is shown.

To confirm the effect of Al and Fe on the STOP1 pathway, RT-PCR was performed for two direct targets genes of STOP1: *ALMT1* and *CIPK23* (Figure 5A). WT seedlings were grown for 10 days on a rich-phosphate medium and transferred 24 h on low-phosphate media supplemented with Fe or Al conditions described in the figure. The roots were harvested for RNA extraction. Concerning *CIPK23* expression, whatever the conditions tested, the *CIPK23* expression remained stable. Instead, the *ALMT1* relative expression tends to show an upregulation in presence of Al alone and with Fe. Surprisingly, a low tendency was observed in the presence of Fe alone. No statistical differences could be observed using the multiple comparison Kruskal-Wallis test applied with Dunn correction, however, these tendencies were observed within 4 independent experiments (data not shown). In Supplementary Figure 4, we observed the *ALMT1* pattern induced by the Al, Fe independently and combined stresses by transferring 4 days old WT plants on the indicated media for 24 h. The Al conditions show an *ALMT1* induction in the whole root, that is coherent with the RT-PCR results in Figure 5A. The combined Fe, Al condition shows a localized upregulation in the root tip that is also visible through the RT-PCR. To note, this stress induces a strong root growth arrest characterized by a bulge of the root tip in accordance with Figure 4C. However, without Al, the Fe_10_ condition induces *ALMT1* in the root tip only display a low but not significant induction by RT-PCR (Figure 5A). One explanation could be that the expression of *ALMT1* is more localized at the root tip than in the rest of the root, and taking the whole root for RT-PCR dilute its signal.

Thus, to assess the effect of these stresses on the STOP1 abundance in the root tip specifically, we used plants carrying the construct *pSTOP1:GFP-STOP1* to measure the accumulation of STOP1 at the nuclei through GFP fluorescence. In Figure 5B, 5 days old plants were transferred 4 h on the indicated media before confocal observation. As positive control, the addition of 5 μM AlCl_3_ shows an accumulation of GFP at the nuclei. Increasing doses of FeCl_2_ from 10 to 80 μM were added to the media tends to show a correlated GFP accumulation at the nuclei. In Fe_50_Al_5_ and Fe_80_Al_5_ conditions, the GFP intensity is higher than in their respective control, Fe_50_, Fe_80_, and Al_5_ and tend to show a synergistic effect between Fe and Al.

Altogether these results show that Fe alone triggers a STOP1 dependent primary root growth arrest at a specific concentration Fe_10_ in the WT. In this condition, *ALMT1* is induced in the root tip, however, no STOP1 accumulation was detected in this condition. Instead, the STOP accumulation was observed in correlation with the increasing dose of Fe in the media. Furthermore, we observed that the addition of low Al concentration (Al_5_) in the media strengthens the Fe-dependent root growth arrest and the *ALMT1* induction in the root tip. Furthermore, the combined stress of Fe and Al is the only condition able to trigger an upregulation of *ALMT1* in *stop1*^33^. Finally, Al interacts with Fe signal pathway on STOP1 by displaying a synergistic effect on STOP1 accumulation when high doses of Fe are added to the media.

## Discussion

The use of agar containing low element contents (P, Fe, Al) allowed us to dissect precisely the effect of acidic pH, Al, and Fe independently on the STOP1 signaling pathway. We used the *pALMT1:GUS* reporter, regulated directly by STOP1 to monitor the detection of distinct environmental signals Fe, Al, and H^+^. First, using this reporter, we observed that seedlings could detect Al at a concentration as low as 2 μM within the media at pH 5.7. Moreover, they also detect 20 μM AlCl_3_ at pH > 6.0 (Figure 1), meaning that plants can detect Al at pH higher than in most studies (pH 4.5–5). Previously in the literature, Martin (1991) showed that the fraction of Al^3+^ at pH > 5.7 is lower than other Al species at this pH. This is coherent with the simulation of Al speciation performed in this study. Further, we observe that at pH 5.7, raising the concentration of AlCl_3_ results in the increase of Al_13_ and in the reduction of the proportion of other Al species, whose concentration remains below 4 μM (Supplementary Figure 1B). From this graph, it is difficult to infer which Al species could specifically activate the STOP1 signaling. The simulation of Al speciation performed on the nutrient solution indicates that 20 μM AlCl_3_ at pH 5.7 should have blocked the root growth of the seedlings due to the abundance of the toxic polynuclear Al_13_. As Al toxicity is mostly observed at acidic pH, where Al^3+^ form is largely predominant and that this form decreases with the increasing pH, it could be hypothesized that STOP1 responds to Al^3+^ specifically. However, the speciation simulation does not allow us to determine whether a particular form of Al activates the STOP1-ALMT1 pathway. It is largely accepted that Al^3+^ is the most abundant form at acidic pH and results in plant toxicity. Nevertheless, we observed that Al_13_ is also an abundant fraction of Al at acidic and neutral pH, thus it is tempting to say that other forms could also be able to activate the STOP1-ALMT1 pathway. No Al-dependent toxicity symptoms on agar plates, assessed through the primary root response of the *stop1* Al-sensitive mutants were observed in our control conditions without or low Al (Figure 2E). As the effect of agar on Al speciation could not be simulated, one hypothesis is that some components of the agar prevent the toxicity of Al_13_, Al^3+^, and maybe other Al species. Consequently, it is tempting to say that the concentration of the bioavailable Al^3+^ in our culture conditions at pH 5.7 remains low and that the use of the *pALMT1:GUS* construct is sensitive enough to detect the presence of tiny amounts of bioactive Al (Figure 2A). Further, a low amount of Al triggers STOP1 accumulation in the nuclei (Figures 2C,D), but does not affect the primary root response of the Al-hypersensitive *stop1* mutants at a concentration below 50 μM AlCl_3_ (Figure 2E). This enabled us to uncouple the detection of Al upstream STOP1, from its toxicity prevented by the STOP1-ALMT1 Al-tolerance mechanism. The identification of this undetermined boundary would allow us to refine the design of Al-focused studies. As reviewed in Bojórquez-Quintal et al. (2017), Al was also observed to have beneficial effects in other species [i.e., plant growth stimulation (Osaki et al., 1997), promotion of other nutrient uptake (Xu et al., 2016)] or toxic effects that are largely described in the literature. The uncoupling of Al signal pathway from its toxicity could allow us to determine the conditions on which the positive effect of Al could be studied.

Furthermore, the Al signal pathway is not the only one to overlap with Al toxicity. As reviewed in Shavrukov and Hirai (2016) H^+^ and Al trigger distinct mechanisms that are important to assess separately. However, as the fraction of soluble Al^3+^ decreases when pH increases, most studies assessing Al stress are performed at pH below 5, thus in conditions where both stresses are overlapping. As mentioned in the introduction, STOP1 was firstly identified as a gene required for proton tolerance (Iuchi et al., 2007). Thus, based on a similar system, the combination of *pALMT1:GUS* as a reporter of STOP1 activity and an Al-poor agar enabled us to show that STOP1 can be induced by H^+^ and Al independently (Supplementary Figure 3). The STOP1-ALMT1 pathway can be activated by Al and H^+^. However, the use of conditions (here P_10_ or P_500_, pH 5.7 without the addition of Fe or Al) in which *ALMT1* is not induced at pH where Al could be bioavailable permitted a fine evaluation of the applied stress. It is fair to say that the absence of *ALMT1* expression in WT can be used as negative control and that these steady states vary according to respective working conditions. Nevertheless, it underlines the importance to distinguish between Al and H^+^ signal pathways and provides one way to monitor them by characterization of one after the other.

We previously showed that the growth inhibition in low phosphate depends on malate exudation by ALMT1 (Balzergue et al., 2017). Fe and Al separately trigger STOP1 accumulation at the nuclei (Godon et al., 2019; Figures 2C,D). The *ALMT1* expression is induced in the root tip when only 10 μM FeCl_2_ is added (Figure 4B and Supplementary Figure 4), inducing the inhibition of the primary root growth at this specific concentration (Figure 4A). To note, no *ALMT1* upregulation was observed using RT-PCR (Figure 5A). The signal triggered by iron at this concentration is localized in the root tip, while the entire roots were used for the RT-PCR, which could have diluted the signal.

Fe and Al ions response mechanisms affect differentially the STOP1-ALMT1 pathway in terms of *ALMT1* expression level (Figures 2B, 5A) but also localization (Supplementary Figure 4) and primary root response (Figures 2E, 4A). Moreover, Fe and Al’s respective signal pathways, when occurring simultaneously, seem to affect each other. Al strengthens the Fe inhibition and the *ALMT1* expression in the root tip. When Al concentration increases in presence of 10 μM FeCl_2_ (Figure 4D) a strong GUS staining is observed in *stop1*^33^ mutant (Figure 4E), which results in a Fe-dependent root inhibition compared to the *stop1*^127^ null allele (Figure 4F). However, Al and Fe separately are not able to trigger *ALMT1* expression in *stop1*^33^ leaky allele root tip, suggesting a synergistic interaction between these two signals on *ALMT1* expression.

To note, the combination of Fe and Al results in strong primary root inhibition in WT (Figures 4C,F) compared to the Fe dose-curve (Figures 2E, 4A) and that the release of the observed inhibition is suppressed in the combined stress condition. Such a similar partial reversion of the inhibition by Al and Fe suggests a common, unknown mechanism. In Figure 4E, the *stop1*^33^ leaky mutant displays an intermediate inhibition compared to *stop1*^127^ null allele. This result is in concordance with (Balzergue et al., 2017) that showed that growth inhibition in a low-phosphate condition depends on malate exudation by ALMT1. Furthermore, a synergistic effect is observed on STOP1 accumulation in the nuclei when both stresses are added in high Fe concentrations (Figure 5B). Surprisingly, no or low accumulation of GFP was observed at low Fe concentration.

Although it would be interesting to test whether Fe can mimic Al on ALMT1 protein, our results suggest it is not the case because the dose-response curves with Fe are substantially different when there is Al (Figure 4C) or not in the growth media (Figure 4A). Supplementing the medium with Al profoundly changes the root growth response to Fe.

To note, the GUS staining is a sensitive marker that qualitatively reflects the activity of the gene transcription promoter resulting in *ALMT1* expression. The GUS system does not infer about any negative transcriptional regulation. Instead, the use of the *pSTOP1:GFP-STOP1* construct reports a balance between protein accumulation and degradation. Therefore, it is not surprising to observe differences in information obtained from the GUS and GFP-STOP1 systems due to their respective reporter activities.

This result has practical consequences for studies about Fe, phosphate, and the STOP1 pathway. Another explanation for the partial reversion of the inhibition by Al and Fe is that STOP1 activates the expression of genes coding for cell wall remodeling enzymes (Sawaki et al., 2009) and the low-phosphate condition also changes the expression of cell wall-modifying enzymes, in particular, some that modify pectins (Hoehenwarter et al., 2016). On the other hand, both Al and Fe are known to accumulate and bind to negatively charged cell wall components such as pectin and hemicellulose (Horst and Wang, 2010; Kochian et al., 2015; Kopittke et al., 2016; Curie and Mari, 2017). One hypothesis could be that intermediate concentrations of Al and Fe interfere with the cell wall remodeling consequences of the Fe- and LPR1-dependent inhibition of root growth under low phosphate conditions.

How Al and Fe activate STOP1 remains to be discovered. We know that the ubiquitin ligase RAE1 and RAH1 and the SUMO E3 ligase SIZ1 destabilize the STOP1 protein (Zhang et al., 2019; Fang et al., 2021a,b; Xu et al., 2021), but we do not know whether Fe and Al act upstream of these two enzymes. It is unlikely that these two metals act directly on them, as they do not appear to have a Fe-binding domain. However, it is conceivable that the sensing of Fe and Al occurs upstream or in cooperation with these enzymes.

This work now shows that Al strengthens the Fe-dependent root growth response at all Fe concentrations and that this interference with the Fe response depends on STOP1-ALMT1 signaling. This suggests that both Fe and Al signal pathways can interact with *ALMT1* expression level and spatial regulation (Figure 5A and Supplementary Figure 4). In addition, recent structural characterization of Arabidopsis ALMT1 showed that Al binds the extracellular side and activates the opening of this channel (Wang et al., 2021). Together, the Al inducing *ALMT1* expression through STOP1 accumulation and Al activating ALMT1 activity promote higher exudation of malate. Another hypothesis is that the presence of Al and Fe in the media could enhance the root growth inhibition, probably by catalyzing ROS production (Muller et al., 2015; Balzergue et al., 2017; Mora-Macias et al., 2017; Zheng et al., 2019). In a first study (Ruipérez et al., 2012), Al was found to have a pro-oxidant effect by promoting the Fenton reaction, however, a second computational study focused on citrate (Mujika et al., 2018) showed that in one hand, when Al is chelated to citrate it can have a pro-oxidant effect by stabilizing aluminum superoxide complexes but on the other hand, the chelation of Fe by citrate prevents the Fenton reaction. Thus, the authors concluded that citrate can have both promotor and protective roles in the Fenton reaction depending on multiple factors, such as the initial concentration of elements. This could explain the strengthening effect of Al but also the relieving effect on root growth observed in the dose-response curves at specific concentrations (Figures 4A,C,F).

In summary, firstly we identified a system based on a well-characterized agar containing low amounts of P, Fe, and Al combined with the *pALMT1:GUS* reporter gene that allowed us to uncouple Al signal pathway from its toxicity. We also observed that H^+^ and Al independently activate the STOP1-ALMT1 pathway, while both stresses are usually overlapping in experimental settings. Lastly, our results show a close intertwining of the effects of Fe and Al on the plant *via* the STOP1 signaling pathway. Al strengthens the Fe response on plant growth but also on *ALTM1* expression in the root tip. Carefully distinguishing the role of Fe and Al in an experimental setup will help to discover how plants sense Fe and Al in the STOP1 signaling pathway.

## Data Availability Statement

The original contributions presented in the study are included in the article/Supplementary Material, further inquiries can be directed to the corresponding author/s.

## Author Contributions

LL, CM, and TD designed the experiments, analyzed the data, and revised the manuscript. LL performed most of the experiments. LF performed simulations of Al speciation. ND gave technical support for RT-PCR and took care of plants. PD assisted with RT-PCR. TD supervised and coordinated the project, carried out the dose-response curve with iron. TD and LL wrote the manuscript and prepared the figures. SP participated in the funding of CM. LN made valuable comments on the project. All authors contributed to the article and approved the submitted version.

## Conflict of Interest

The authors declare that the research was conducted in the absence of any commercial or financial relationships that could be construed as a potential conflict of interest.

## Publisher’s Note

All claims expressed in this article are solely those of the authors and do not necessarily represent those of their affiliated organizations, or those of the publisher, the editors and the reviewers. Any product that may be evaluated in this article, or claim that may be made by its manufacturer, is not guaranteed or endorsed by the publisher.

## Footnotes

1
https://www.zeiss.com/

## Supplementary Material

Supplementary Figure 1 **(A)** Four day-old *stop1*^127^ null mutant seedlings (carrying the *pALMT1:GUS* reporter) were grown on phosphate-rich medium and transferred on the indicated P_10_Fe_0_, pH 5.7 medium supplemented with the indicated concentrations of AlCl_3._ After 3 days a GUS staining was performed on roots. Three representative root tips are shown per condition. **(B)** Al induced *ALMT1* from hydroponic culture medium. Plants were grown in hydroponic in MS/10 solution at pH 5.7 in phosphate-rich condition (P_500_) for 3 days. Seedlings were transferred 12 h in 30 mL volume in presence of absence of 20 μM AlCl_3_ before GUS staining for 45 min. Scale, 500 μm.

Supplementary Figure 2 Speciation of aluminum in the growth media. The speciation of Al was simulated (see section “Materials and Methods”) for the growth media used in this work, but without agar and the MES and HomoPipes buffers. **(A)** Simulation for a medium supplemented with 20 μM AlCl_3_, at the indicated pH. The% are counted in relation to the quantity of Al. Note the dominance of Al^3+^ at pH below 5.1, the dominance of polynuclear species Al_13_ between pH 5.1 and 6.5, and the dominance of Al(OH)4- and Al(OH)3(aq) above pH 6.1. **(B)** Simulation for a growth medium at pH 5.7 supplemented with 2, 5, 10, 20, 40, 50, 80, or 200 μM AlCl_3_. Note the dominance of mononuclear Al hydroxyl species at low concentrations of Al, and the dominance of the polynuclear species Al_13_ above 10 μM. Inset: corresponding concentrations of Al species (log-log axes).

Supplementary Figure 3 *ALMT1* is expressed under acidic pH independently from the presence of Al on agar medium. 4 days old WT seedlings carrying the *pALMT1:GUS* construct were grown on phosphate-rich medium (P_500_) in the low-agar content at the indicated pH before GUS staining. Scale, 500 μm.

Supplementary Figure 4 Spatial *ALMT1* expression pattern in the root tip. 4 days old WT seedlings carrying the *pALMT1:GUS* construct were grown on phosphate-rich medium (P_500_) in the low-agar content at pH 5.7 and transferred 24 h on the indicated condition with low-phosphate content (P_10_) before GUS staining for 1 h.
