# Supplementary figures and images for: Uncoupling Aluminum Toxicity From Aluminum Signals in the STOP1 Pathway
Source: Front Plant Sci. 2022 May 3;13:785791. doi: 10.3389/fpls.2022.785791 (PMC9111536; doi:10.3389/fpls.2022.785791)

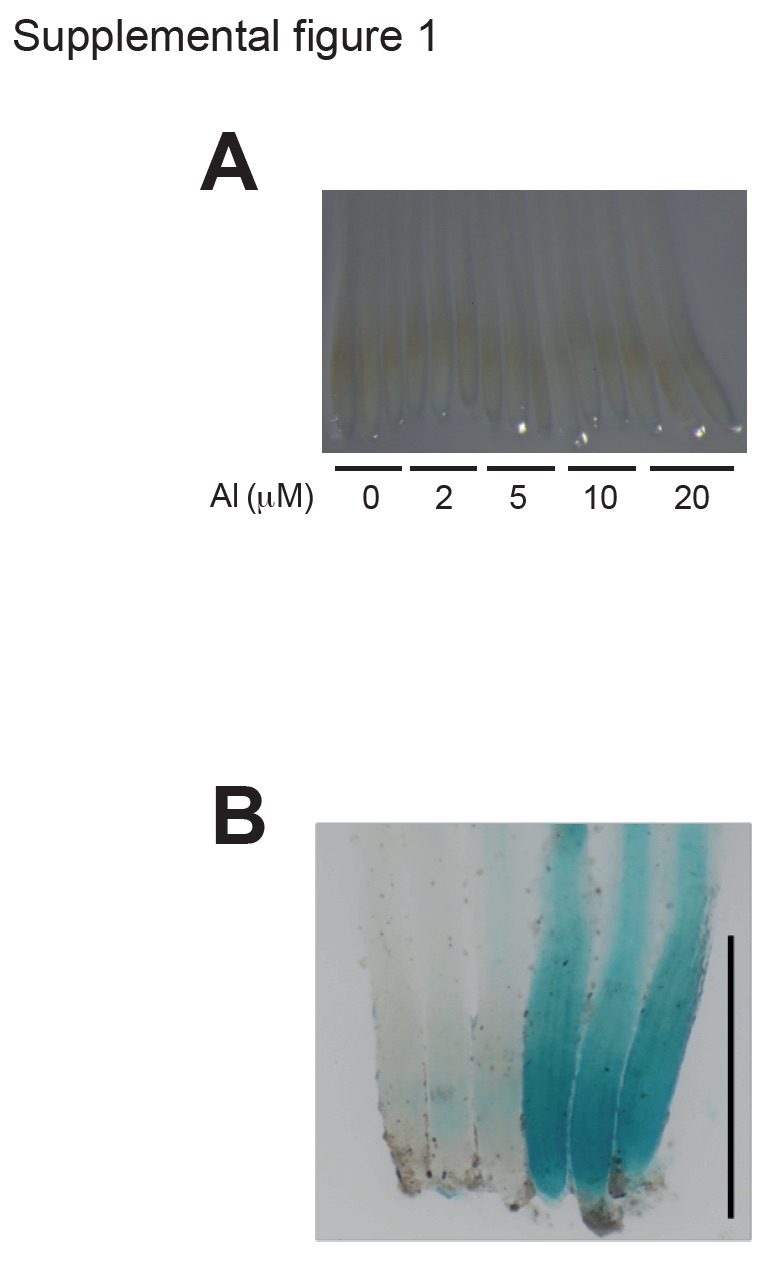

Supplement: Supplementary Figure 1 — (A) Four day-old stop1127 null mutant seedlings (carrying the pALMT1:GUS reporter) were grown on phosphate-rich medium and transferred on the indicated P10Fe0, pH 5.7 medium supplemented with the indicated concentrations of AlCl3. After 3 days a GUS staining was performed on roots. Three representative root tips are shown per condition. (B) Al induced ALMT1 from hydroponic culture medium. Plants were grown in hydroponic in MS/10 solution at pH 5.7 in phosphate-rich condition (P500) for 3 days. Seedlings were transferred 12 h in 30 mL volume in presence of absence of 20 μM AlCl3 before GUS staining for 45 min. Scale, 500 μm. [file Image_1.JPEG]

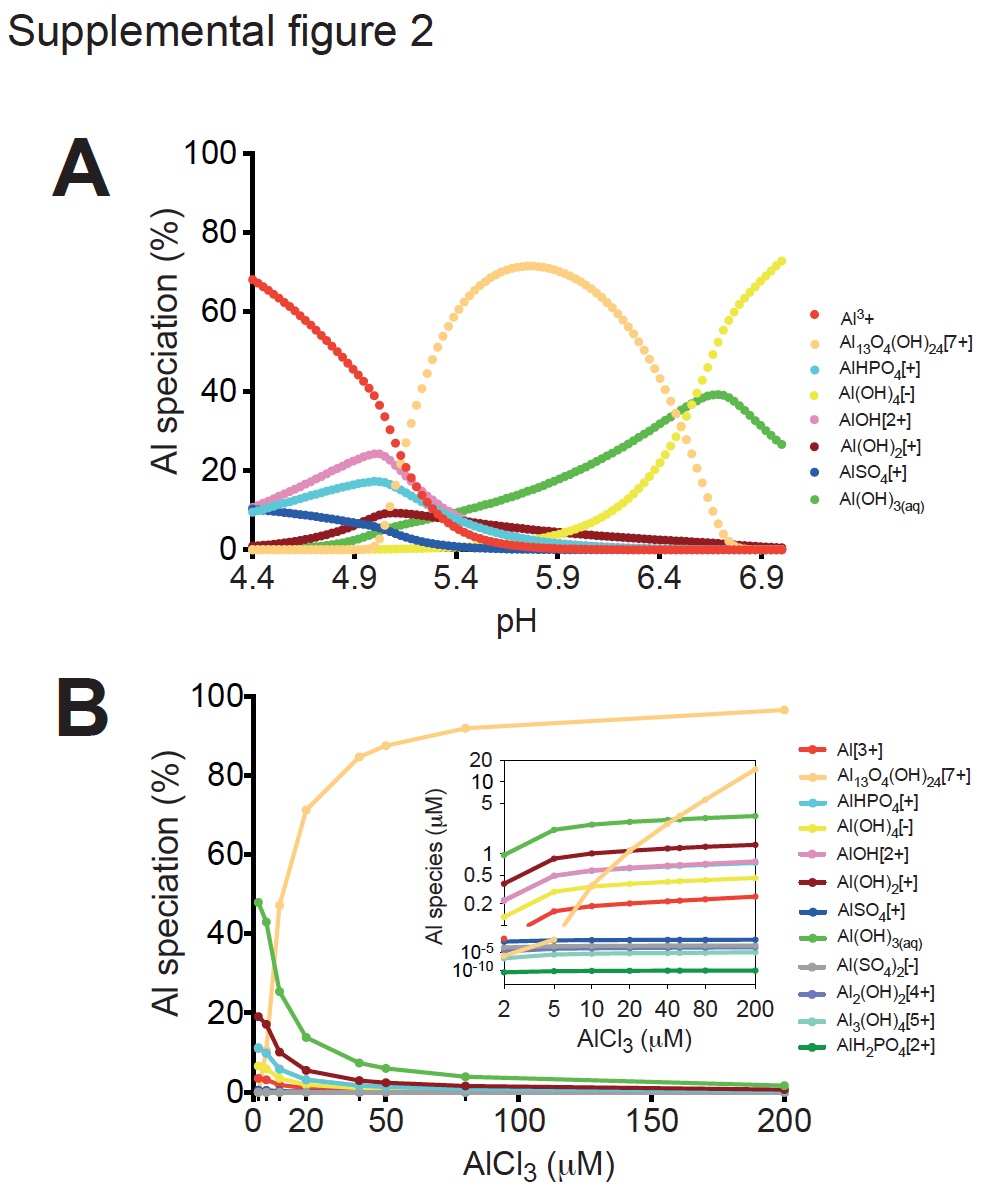

Supplement: Supplementary Figure 2 — Speciation of aluminum in the growth media. The speciation of Al was simulated (see section “Materials and Methods”) for the growth media used in this work, but without agar and the MES and HomoPipes buffers. (A) Simulation for a medium supplemented with 20 μM AlCl3, at the indicated pH. The% are counted in relation to the quantity of Al. Note the dominance of Al3+ at pH below 5.1, the dominance of polynuclear species Al13 between pH 5.1 and 6.5, and the dominance of Al(OH)4- and Al(OH)3(aq) above pH 6.1. (B) Simulation for a growth medium at pH 5.7 supplemented with 2, 5, 10, 20, 40, 50, 80, or 200 μM AlCl3. Note the dominance of mononuclear Al hydroxyl species at low concentrations of Al, and the dominance of the polynuclear species Al13 above 10 μM. Inset: corresponding concentrations of Al species (log-log axes). [file Image_2.JPEG]

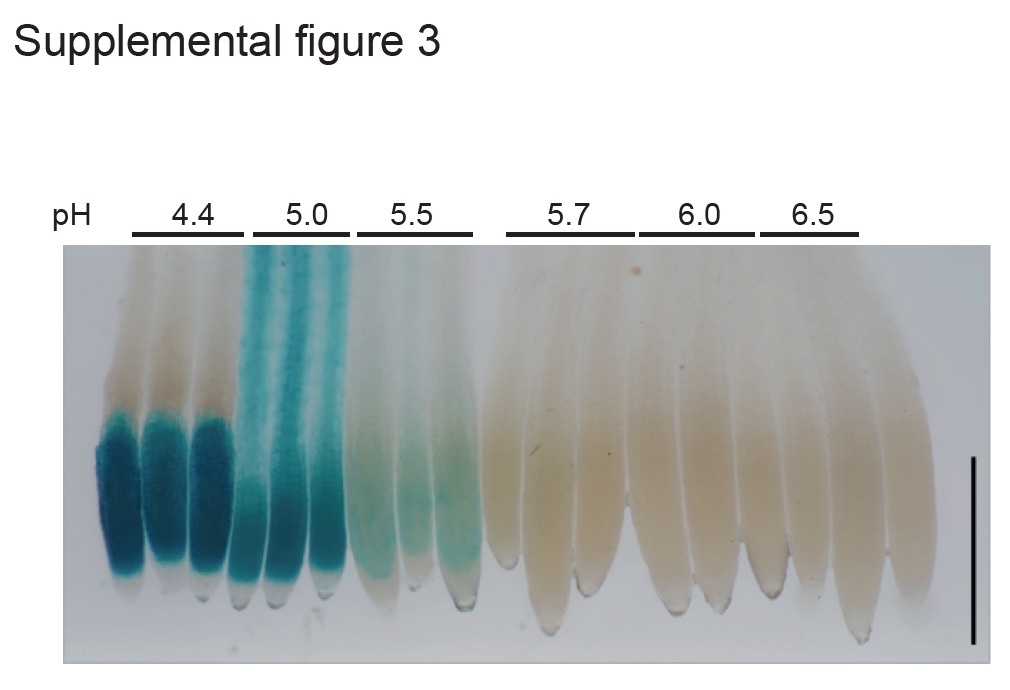

Supplement: Supplementary Figure 3 — ALMT1 is expressed under acidic pH independently from the presence of Al on agar medium. 4 days old WT seedlings carrying the pALMT1:GUS construct were grown on phosphate-rich medium (P500) in the low-agar content at the indicated pH before GUS staining. Scale, 500 μm. [file Image_3.JPEG]

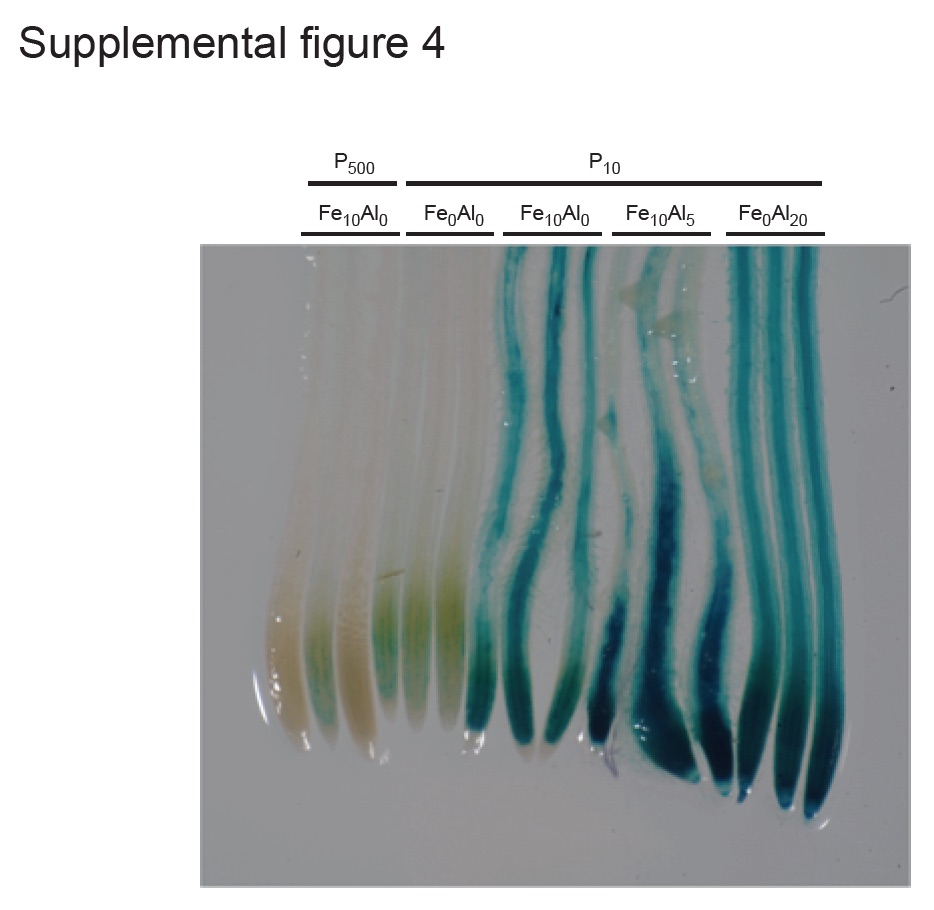

Supplement: Supplementary Figure 4 — Spatial ALMT1 expression pattern in the root tip. 4 days old WT seedlings carrying the pALMT1:GUS construct were grown on phosphate-rich medium (P500) in the low-agar content at pH 5.7 and transferred 24 h on the indicated condition with low-phosphate content (P10) before GUS staining for 1 h. [file Image_4.JPEG]
