# Supplementary material for: Uncoupling Aluminum Toxicity From Aluminum Signals in the STOP1 Pathway
Source: Front Plant Sci. 2022 May 3;13:785791. doi: 10.3389/fpls.2022.785791 (PMC9111536; doi:10.3389/fpls.2022.785791)
Supplement: Supplementary file 5 [file Data_Sheet_1.docx]

**Supplementary Table 1**. Equilibrium reactions used for speciation simulation of Al

| Equilibrium reactions | Equilibrium constants (log K) |
| --- | --- |
| Al^3+^ + H_2_O = AlOH^2+^ + H^+^ | -5.00 |
| Al^3+^ + 2H_2_O = Al(OH)_2_^+^ + 2H^+^ | -10.34 |
| Al^3+^ + 3H_2_O = Al(OH)_3(aq)_ + 3H^+^ | -15.60 |
| Al^3+^ + 4H_2_O = Al(OH)_4_^-^ + 4H^+^ | -22.20 |
| 2Al^3+^ + 2H_2_O = Al_2_(OH)_2_^4+^ + 2H^+^ | -7.69 |
| 3Al^3+^ + 4H_2_O = Al_3_(OH)_4_^5+^ + 4H^+^ | -13.88 |
| 13Al^3+^ + 28H_2_O = Al_13_O_4_(OH)_24_^7+^ + 32H^+^ | -98.73 |
| Al^3+^ + SO_4_^2-^ = AlSO_4_^+^ | 3.01 |
| Al^3+^ + 2SO_4_^2-^ = Al(SO_4_)_2_^-^ | 4.9 |
| Al^3+^ + HPO_4_^2-^ = AlHPO4^+^ | 7.4 |
| Al^3+^ + H^+^ + HPO_4_^2-^ = AlH_2_PO_4_^2+^ | 3.1 |
| Al^3+^ + 3H_2_O = Al(OH)_3_ + 3H^+^ | -6.97 |
| 3Al^3+^ + K^+^ + 2SO_4_^2-^ + 6H_2_O = KAl_3_(SO_4_)_2_(OH)_6_ + 6H[^+^] | 2.40 |

**Supplementary Table 2. RT-PCR primers sequences**

| Gene | ATG number | sequences |
| --- | --- | --- |
| *ALMT1* | AT1G08430 | F: GGCAGTGTGCCTACAGGATT  R : CGATTCCGAGCTCATTCTTC |
| *CIPK23* | AT1G30270 | F: CGTTTTGGAATTCGTCACTG  R: TGTTGGAAATACTTCCTCGC |
| *Tubulin* | AT5G62690 | F: GAGCCTTACAACGCTACTCTGTCTGTC  R: ACACCAGACATAGTAGCAGAAATCAAG |
